# Bio-Inspired Hierarchical Carbon Nanotube Yarn with Ester Bond Cross-Linkages towards High Conductivity for Multifunctional Applications

**DOI:** 10.3390/nano12020208

**Published:** 2022-01-10

**Authors:** Sidra Saleemi, Mohamed Amine Aouraghe, Xiaoxiao Wei, Wei Liu, Li Liu, M. Irfan Siyal, Jihyun Bae, Fujun Xu

**Affiliations:** 1 Key Laboratory of Textile Science & Technology, Ministry of Education, College of Textiles, Donghua University, Shanghai 201620, China; sidraatif.tet@pu.edu.pk (S.S.); med.aouraghe@gmail.com (M.A.A.); 1189138@dhu.edu.cn (X.W.); liliull@dhu.edu.cn (L.L.); 2 Institute of Polymer and Textile Engineering, University of the Punjab, Quaid-e-Azam Campus, Lahore 54590, Pakistan; 3 School of Textiles and Fashion, Shanghai University of Engineering Science, Shanghai 201620, China; littlewand@163.com; 4 Department of Textiles and Clothing, National Textile University, Karachi Campus, Karachi 74900, Pakistan; irfan.tex@gmail.com; 5 Human-Tech Convergence Program, Department of Clothing and Textiles, Hanyang University, Seoul 04763, Korea

**Keywords:** bio-inspired, carbon nanotube yarn, esterification, microstructures, electrical properties, smart materials

## Abstract

The cross-linked hierarchical structure in biological systems provides insight into the development of innovative material structures. Specifically, the sarcoplasmic reticulum muscle is able to transmit electrical impulses in skeletal muscle due to its cross-linked hierarchical tubular cell structure. Inspired by the cross-linked tubular cell structure, we designed and built chemical cross-links between the carbon nanotubes within the carbon nanotube yarn (CNT yarn) structure by an esterification reaction. Consequently, compared with the pristine CNT yarn, its electrical conductivity dramatically enhanced 348%, from 557 S/cm to 1950 S/cm. Furthermore, when applied with three voltages, the electro-thermal temperature of esterified CNT yarn reached 261 °C, much higher than that of pristine CNT yarn (175 °C). In addition, the esterified CNT yarn exhibits a linear and stable piezo-resistive response, with a 158% enhanced gauge factor (the ratio of electrical resistance changing to strain change ~1.9). The superconductivity, flexibility, and stable sensitivity of the esterified flexible CNT yarn demonstrate its great potential in the applications of intelligent devices, smart clothing, or other advanced composites.

## 1. Introduction

The aerogel-spun CNT fiber/yarn fabricated by floating catalyst chemical vapor deposition (FCCVD) is a pure CNT assembly with a one-dimensional fiber-like structure [1]. By inheriting the characterizations of individual CNTs, CNT yarn shows a variety of advantages of high tensile strength, extraordinary structural flexibility, and outstanding corrosion/oxidation resistivity [2,3,4], which give it great potential to be applied in the field of high-performance fibers such as high strength conductive fibers, energy harvesting/storage fibers, flexible medical/bio-devices, artificial muscles, and wearable electronics, to name a few [3,5,6,7,8,9]. Currently, nanostructures have found applicability in different areas of research investigations [10]. The aerogel-spun CNT fiber/yarn fabricated by floating catalyst chemical vapor deposition (FCCVD) is a pure CNT assembly with a one-dimensional fiber-like structure. However, the intrinsic weak Van der Waals interactions between CNTs render a rough challenge in attaining the advantage of the individual CNT properties [11,12]. Furthermore, the Van der Waals forces are significantly dominated by the contact areas between the adjacent CNTs. Therefore, the porous microstructure inside CNT fiber hinders the strong inter-bundle junctions and entanglements, resulting in relatively low structural stability and limited electrical conductivity.

To combat this issue, scholars have tried various methods to obtain stronger inter-tube coupling and enhance the electrical conductivity of the CNT yarn. These methods could mainly be classified into three categories: firstly, physical treatments through the densification process, including twisting [13,14], cyclic stretching [15], rolling, or capillary force condensing [16]; secondly, chemical treatments by chemical solvents, wet cryogenic modification, annealing (high temperature in the air), and modification of CNTs by polymer [17,18,19,20,21,22,23,24]; and thirdly, the plasma treatments to build strong inter tube couplings [25,26]. The densification strategy by chemical treatments can effectively compact the CNTs/bundles and potentially enhance the contact area between neighboring CNTs [27]. However, the week electron transfer through Van der Waals interaction only leads to the limited increase in conductivity of the physically treated CNT yarn. For the chemical treatments, the interactions between the CNTs could be enhanced via creating covalent or hydrogen bonds between the tubes through functionalization [28,29] and esterification of CNTs as reported by Yong-O Im, focusing on improving the strength of CNTs [30]. However, this study was conducted on CNT fiber instead of yarn, and this work was not extended to its conductive properties for smart textile applications. Besides the chemical treatments, the functionalized CNTs can be obtained through irradiation of CNTs, or plasma treatment. Although CNT functionalization provides an effective approach for improving the interfacial interaction, however additional defects of the conversion of sp^2^ into sp^3^ carbon atoms on the CNT surfaces through acid treatment decrease the mechanical and electrical properties of the CNTs yarns. 

To address the aforementioned challenges, herein, we look forward to exploiting a naturalistic assembly existing in the human body [15,31]. There are many reports on cross-linked CNT fibers; however, this study reports cross-linking of CNTs toward CNT yarn. Moreover, cross-linking of CNT yarn using acids with bio-based cross-linker, i.e., 1, 5-pentanediol have not been elaborated before for conductive properties and its application. Inspired by the bridging mechanism in sarcoplasmic reticulum muscles, we manufactured the cross-linked hierarchical structured CNT yarn by esterifying the CNT film using a bio-based cross-linker and then twisting it into a yarn. The esterified CNT yarn with ester bond cross-linkages exhibited simultaneously enhanced electrical, electro-thermal, and strain sensing properties. The characterizations of the esterified CNT yarn were identified by using SEM, Raman spectroscopy, FTIR, and XPS. To the best of our knowledge, a novel esterified CNT yarn with a diameter of 150 µm with such high conductive properties has not yet been studied; however, highly conductive yarns and fibers are manufactured by using different modification techniques with very fine diameters ranging 50–20 µm, which are difficult to handle during weaving and knitting of conductive yarn [32]. To verify the functions of the esterification, the acidification of CNT yarn was also conducted for further comparison. 

## 2. Materials and Methods

### 2.1. Materials

The MWCNTs (Multiwall Carbon Nanotubes) with 20 walls and 25 nm average diameter were fabricated in CNT film by floating chemical vapor deposition by Suzhou Institute of Nano-Tech and Nano-Bionics, Chinese Academy of Sciences, Hangzhou, China. HNO_3_ (concentrated nitric acid 65–68%) and H_2_SO_4_ (concentrated sulfuric acid 95.0–98.0%), bio-based alcohol, i.e., 1, 5-pentanediol were supplied by Hangzhou Meite Industry Co., Ltd. (Hangzhou, China).

### 2.2. CNT Yarn Fabrication and Esterification 

Prior to surface treatment, CNT film was cut by using a sharp razor blade measuring 2 mm. Chemical modification of CNTs was achieved by dipping the CNT film in a solution of HNO_3_ (concentrated nitric acid 65–68%) and H_2_SO_4_ (concentrated sulfuric acid 95.0–98.0%) in 1:3, respectively, for one hour. After that, the CNT film was cleaned by using acetone to remove the residual acid. Esterification of CNTs was carried out by soaking CNT film in a solution containing 10 mL 1, 5-pentanediol and 0.1 mL H_2_SO_4_ for 10 min [30]. After, the treated film was gradually cured in a drying oven at 70 °C for one hour and 90 °C for two hours in a vacuum environment. Finally, the dried CNT films were twisted to form a CNT yarn by using with an average diameter of 150 ± 2 µm through an electric motor having a rotational speed of 1200 twist/min.

### 2.3. Property Testing

The morphological features of CNT yarns were evaluated by using SEM (Scanning Electron Microscope, Hitachi, TM3000, and 5 kV, Tokyo, Japan) and FESEM (Field Emission Scanning Electron Microscope, Hitachi, S4800, and 5 kV, Tokyo, Japan). Transmission electron microscopy (TEM JEOLJEM-2100 at 200 kV, Arizona State University, Tempe, AZ, USA) was used to observe the structural changes of individual CNT in CNT yarn. The graphitization of CNTs was assessed by Polarized Raman Spectral (Renishaw in Via Raman microscope, Beijing, China). The CNT yarn samples were analyzed by Fourier transform infrared spectroscopy (FTIR, Spectrum Two, UK) in the range of 4000–500 cm^−1^ and X-ray photoelectron spectroscopy by using (XPS Escalab 250Xi, Shanghai, China). The weight loss of CNT yarn in nitrogen was investigated by the thermal gravimetric analysis (TGA, Netzsch TG 209F1, Germany). The yarn diameters were obtained by using a microscope (ECLIPSE LV100 POL, Nikon Shanghai, China). Electrical properties were evaluated by using a two-probe multi-meter (Agilent 34410A, TX, USA). The strain sensing behavior of the yarn was characterized by recording resistance through a multi-meter along with a controlled strain applied by a tensile tester (XQ-2, Xusai Instrument, Shanghai, China). The electro-thermal properties were determined by the digital multi-meter Keysight 34450A Preetz, Germany. A thermal camera (Fotric 225) with a DC voltage supply was used to record thermography results. 

## 3. Results and Discussion

### 3.1. Hierarchical Structure of the Esterified CNT Yarn

**Figure 1:**
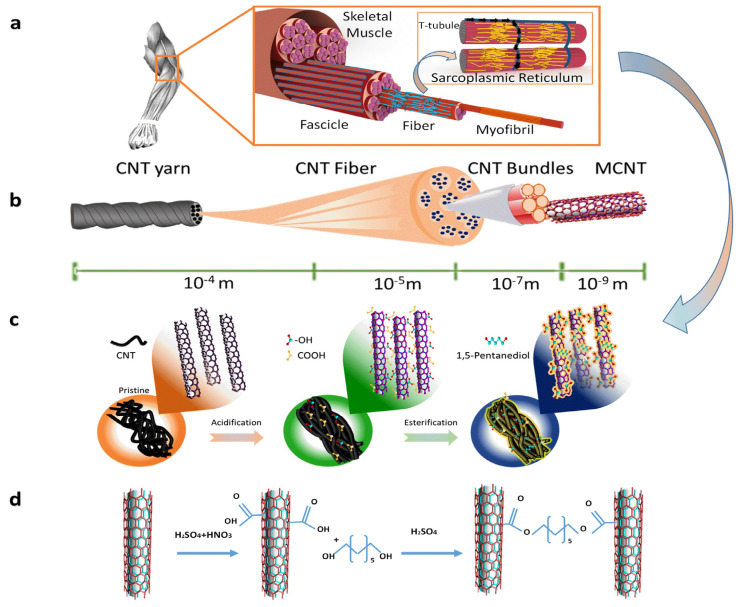
(**a**) Hierarchical structure of skeletal muscle with sarcoplasmic reticulum showing a path for electrons to move through T tubules. (**b**) Schematic demonstration of the hierarchical structure of carbon nanotube presenting CNT yarn, CNT fiber, CNT bundles, and CNTs with their scales. (**c**) Illustration of chemical modification of pristine CNTs towards acidified and esterified structures. (**d**) Chemical reaction between CNTs due to acidification and esterification.

The comparison of the hierarchical structure of skeletal muscle and esterified CNT yarn was shown in Figure 1a,b. In normal muscle cells, myofibrils are long tubular microstructures consisting of thick and thin filaments. These thick and thin filaments not only form cross-bridges to facilitate contraction but also provide an interconnected horizontal network of tubes for the transportation of calcium ions. Emerging strategies to mimic this natural assembly in our research use designed structures incorporated with cross-linking through chemical modification with an aim to form an interconnecting network among CNTs, similar to the sarcoplasmic reticulum network, which can offer multiple positive attributes in terms of electrical performances. Figure 1c explained the schematic of pristine, functionalized CNTs where treatment of acid results in functional groups such as carbonyl group (C=O) and a hydroxyl group (O-H) [33] and finally cross-linked CNTs where covalent bonds were introduced in terms of an ester bond between adjacent CNTs through esterification; while the detailed chemical reaction of esterification is specified in Figure 1d. Furthermore, the existence of multiple CNTs running parallel, making a bundle-like arrangement, signifying muscle cells within muscle tissue, ultimately led to improved conductive properties of the final product. 

**Figure 2:**
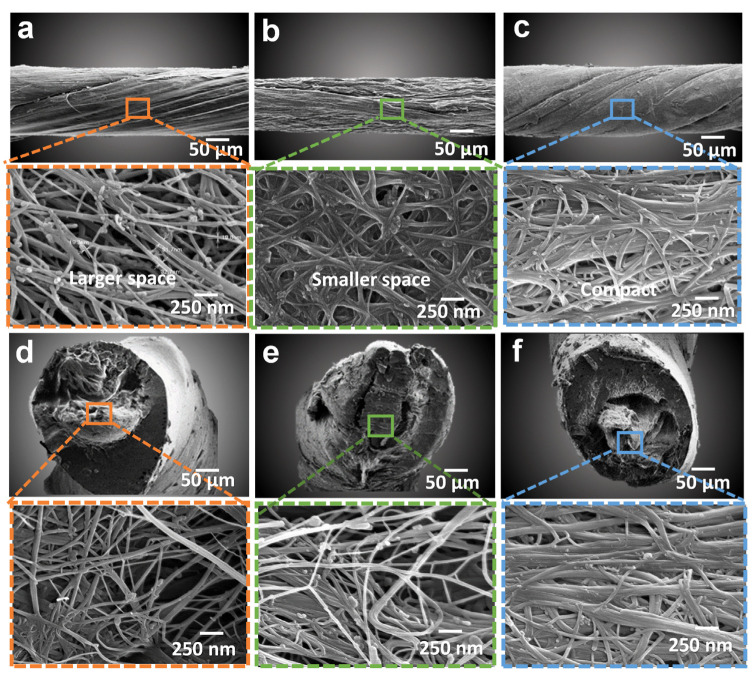
SEM images of (**a**,**d**) Pristine CNT yarn; (**b**,**e**) Acidified CNT yarn; and (**c**,**f**) Esterified CNT yarn in longitudinal and cross-sectional direction, respectively presenting loosely packed to highly dense CNTs structure.

Figure 2a–c (longitudinal view) and Figure 2d–f (cross-sectional view) expressing Scanning Electron Microscope images for pristine, acidified, and esterified CNT yarn correspondingly, showed the comparatively compact structure of CNT bundles after chemical modification. Both longitudinal and cross-sectional views (Figure 2a,d) of pristine CNT yarn showed a highly porous structure that has large inter-tube distances leading to weak Vander Waal forces within pure CNT bundles. On the other hand, acid treated MWCNTs assemblies assisted the CNTs with a carboxylic group in a nondestructive way, as pointed out in Figure 2b,e, which led to removal of unwanted impurities and void spaces leading to more operative electrical properties of chemically modified CNT yarn.

To further elucidate the magnificent effect of esterification, Figure 2c,f revealed a more firmly packed inter-tube structure of these functionalized CNTs having considerably fewer impurities. From the TEM images in Figure S1, it can be observed that a significant amount of amorphous carbon was removed after the acid treatment. The functionalized CNT sample shows a wavy or bumpy and irregular surface, presenting functional groups which could be attributed to the esterification of CNTs [34,35].

### 3.2. Analysis of CNT Yarn Using Raman, FTIR, and XPS

**Figure 3:**
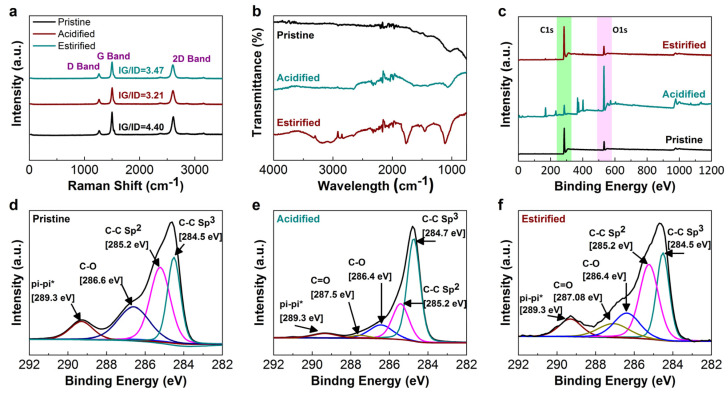
(**a**) Raman Spectroscopy of the D- and G-bands of pristine, acidified, and esterified CNT yarns; (**b**) FTIR of untreated and treated CNT yarns; (**c**) XPS analysis reveals a peak corresponding to C1s at 284.5 eV, N1s at 401.1, and O1s at 532.1 eV. C1s; and XPS spectra for (**d**) pristine, (**e**) acidified, and (**f**) esterified CNT yarns.

Analysis of Raman, FTIR, and XPS of Pristine and treated CNT yarns are shown in Figure 3. The Raman spectra of pristine CNT yarn have shown a sharp peak at 1590 cm^−1^, demonstrating a well-organized structure of CNTs as given in Figure 3a, whereas acid functionalization of CNTs resulted in structural defects on CNTs as carboxylic group influenced the transformation of sp^2^ carbon to sp^3^ carbon [36]. The G peak at ~1580 cm^−1^ was depressed while the D peak at ~1350 cm^−1^ became sharper due to the introduction of functional groups, i.e., hydroxylic and carboxylic groups, shifting the value of IG/ID (intensity ratio of G- and D-band) from 4.4 to 3.21. However, this alteration was increased to 3.47 when CNTs fibers were treated with alcohol for esterification, indicating the unaffected number of sp^3^ carbons in spite of having ester cross-linking within CNTs assembly. 

Furthermore, FTIR spectra were used to confirm the reaction between CNTs within CNT yarns, as revealed in Figure 3b. It is well-identified that pristine CNTs are composed of a highly stable structure without any functional group on CNTs surface, although prominent peaks of functional groups were observed after chemical treatments. The peak at 3410.15 cm^−1^ can be associated with the O-H stretch from carboxyl groups (O=C-OH and C-OH). The peak at 2364 cm^−1^ was also related to the O-H stretch from strongly hydrogen-bonded-COOH [37]. The characteristic peak at (1130 cm^−1^) corresponded to the stretching of the C-O bond in the carboxylic group. The bending and stretching mode of O-H has been observed at 1440–1395 cm^−1^ and 3296–3300 cm^−1^. Prominent C-O and C=O peak of ester groups were observed at 1130 and 1720 cm^−1^, respectively [38,39]. These characteristic peaks indicated that the carboxylic groups and ester bonds were successfully introduced in acidified and esterified yarn. 

Observing XPS spectra of pristine, acidified, and esterified CNT yarns, Figure 3c exposed the increase in the intensity of O1s after the chemical modification of CNTs with acids confirming the presence of functional groups on the surface. The peaks for π–π*, C=O, C-O, sp^2^ carbon, and sp^3^ carbon were perceived at 289.2, 287.5, 286.6–286.4, 285.5–285.2, and 284.9–284.3 eV, respectively, in all the samples of CNTs [40,41,42]. Figure 3d clarifying the C1s nuclear polarography of pristine CNT yarn justifies the stability of CNTs structure with the presence of both sp^3^ and sp^2^ bond for π–π* interaction but without covalent bonding. Figure 3e has revealed the appearance of C=O bonds due to acidification at bonding energy of 287.5, discovering the reduced height of the sp^2^ bond and sharper one for the sp^3^ bond. Lastly, a stable cross-linked structure of assembled CNTs was noticed in Figure 3f due to the enhanced intensity of the sp^2^ bond during esterification. Figure S2 evaluated thermal gravity analysis (TGA) under nitrogen to assess the degradation behavior of CNT yarns for conductive applications. Chemical treatment caused defects (functional groups) on a stable structure of CNTs, thus resulting in early degradation [43]. TGA results verified that pristine CNT yarn remained stable in nitrogen supply up to 800 °C while treated CNT yarn started degradation at 280 °C.

### 3.3. Electrical Properties

**Figure 4:**
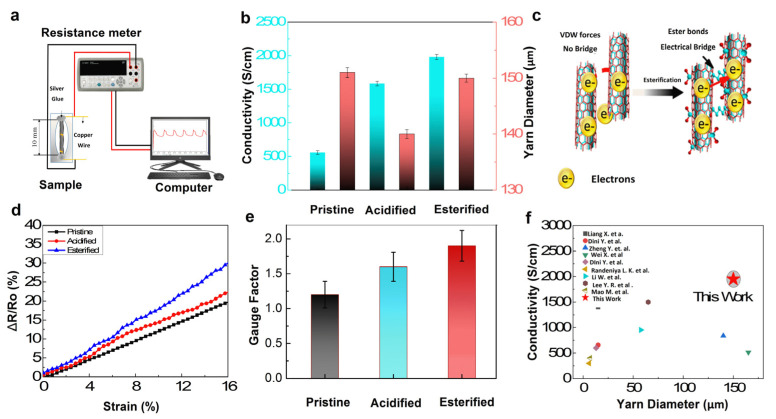
(**a**) Demonstration of CNT yarn specimen and its connection with resistance meter and computer; (**b**) Electrical conductivity and diameter of pristine, acidifed, and esterified CNT yarn; (**c**) Schematic diagram of the flow of electrons in cross-linked CNTs; (**d**) Relative change in resistance due to strain; (**e**) Gauge factor of CNT yarn; and (**f**) Comparison of the electrical conductivity of modified CNT yarn with previous studies.

Electrical properties were evaluated by a two-probe multi-meter (Agilent 34410A, TA, USA). The cross-section ends of the 10 mm CNT yarn sample was connected to the multi-meter using silver paste and copper wire, as demonstrated in Figure 4a,b shows a remarkable boost up of conductivity by 2.8 and 3.5 times, respectively for acidified (1585 S/cm) and esterified (1950 S/cm) CNT yarn than that of untreated CNT yarn (557 S/cm). The cross-section ends of 10 mm CNT yarn sample was connected to the multi-meter using silver paste and copper wire as demonstrated in Figure 4a. The resistance measured by the instrument was used to calculate the electrical conductivity (σ), of CNT yarns by using these formulae:

σ = L/RA (S cm^−1^)

where R, A, and L are the resistance, average yarn cross-sectional area, and length of the specimen.

Similarly, the strain sensing behavior that is GF (Gauge Factor) of CNT yarn was calculated using the formula

$$GF=\frac{\Delta R/Ro}{\Delta L/Lo}$$

where ∆*R* was the resistance change value of the sample (’Ω), *Ro* was the initial resistance value of the sample (’Ω), ∆*L* was the elongation of the sample (mm), and *Lo* was the initial length of the sample.

In general, after functionalization, conductivity decreases due to defects in the form of functional groups. However, here, we are using MWCNTs, and a change in IG/ID was observed, but the conductivity increases due to the presence of functional groups with active sites on CNT, which allow electrons to transfer faster than pristine [44]. Furthermore, cross-linking creates a covalent bridge for electron transfer which enhances the electrical conductivity effectively. The diameter of CNT yarn was also assessed. It was noticed that the diameter of CNT yarn reduced from 151 to 140 µm when treated with acid as it removed impurities and amorphous carbon, which resulted in the densification of CNTs [27]. However, the diameter was increased when it was esterified as acidified CNTs were treated with biopolymer (1, 5-Pentanediol) for esterification, which forms high molecular weight esters, resulting in increases in the diameter of CNT yarn [45,46]. Generally, electron transfer efficiency in CNT yarns significantly depends on interconnections between CNTs, as the inter-tube bridging and junction resistance between CNTs in CNT yarn almost dominate the overall electrical transport. This phenomenon is originated from inter-tube contacts [47]. As mentioned earlier, T-Tubules generates a path for signal transmission. Human muscle is composed of filaments which form and interconnected network of tubes connected by T-Tubules system. Exploiting this muscle arrangement, we postulated to establish an interconnecting network among CNTs, through cross-linking by esterification, which is extremely similar to sarcoplasmic reticulum network as well as beneficial in improving electrical attributes. The chemical modification provided paths for an electron to transfer and reduced the inter-tube contact resistance within CNTs, as illustrated in Figure 4c. In addition, the presence of functional groups reduced contact resistance resulting in greater conductivity in CNT fiber [48]. 

The strain sensing behavior of the yarn was characterized by recording the in situ resistance under controlled strain. Figure 4d, indicating the relationship between relative changes in electrical resistance (∆R/R_0_) due to applied strain (ε) during stretching, discovered the slight changes of piezo-resistive behavior for chemically cross-linked CNT yarn during imposed strain, owing to the minor breakdown of existing conductive paths. Figure 4e showed sensitivity or gauge factor (slope for the curve of the relative change of resistance against applied strain) of pure, acidified, and esterified CNT yarns with a value of 1.2, 1.6, and 1.9, respectively, validating a good yet stable sensing performance of our functionalized and cross-linked CNT yarn. We further compare the electrical conductivity of cross-linked CNT yarns with other literature, as shown in Figure 4f. The as-produced esterified CNT yarn shows an increase up to 1950 S/cm with a diameter of 150 µm, which is higher than the values reported for the modified CNT yarn in other published research works. For instance, Lee Y. et al. reported composite (PVDF/IL) coated nanotubes with multiple twisting strategies results in increased electrical conductivity up to 1500 S/cm [13]. Li W. et al. [16] treated the CNTs with PVA which resulted in improved conductivity up to 447.1 S/cm. Liang X. et al. enhanced the strength, toughness, and electrical conductivity of twist-spun carbon nanotube yarns by p bridging, presenting a CNT yarn with 656.2 S/cm [47]. Randeniya L.K et al. [49], Dini Y. et al. [50], and Miao M. et al. [51] described a modified CNT yarn with 300 S/cm, 600 S/cm, and ~400 S/cm conductivity, respectively. Zheng Y. et al., Dini Y. et al., and Wei X. also reported a CNT yarn with improved electrical properties, i.e., ~1408.1 S/cm, ~400 S/cm, and 514 S/cm, respectively [52,53,54].

### 3.4. Electro-Thermal Properties 

**Figure 5:**
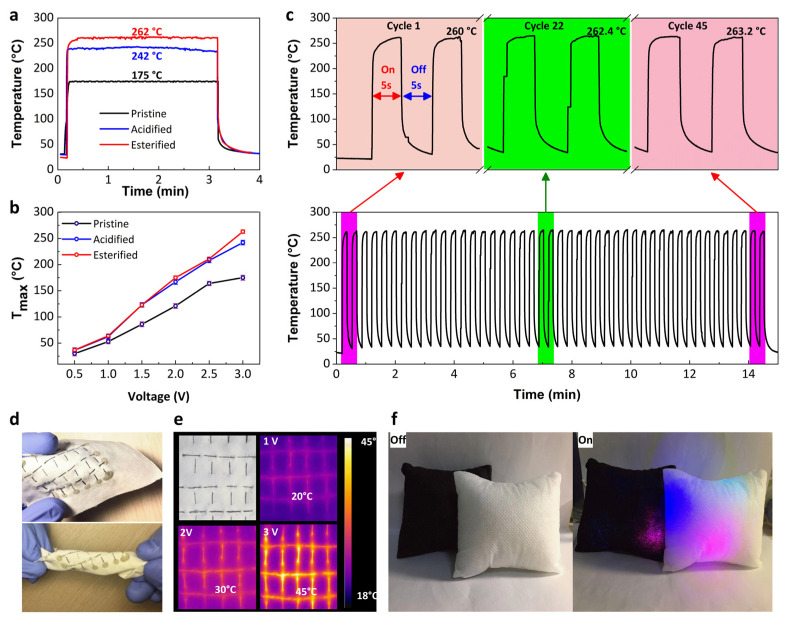
(**a**) E-heating stability of pristine, acidifed, and esterified CNT yarn under 3.0 V; (**b**) Changes of steady-state maximum temperature (T_max_) at different applied voltages; of pristine, acidified, and esterified CNT yarn (**c**) Cyclic E-heating behaviors of Esterified CNT yarn; (**d**) Representation of flexibility of CNT yarn in bent and twisted fabric (**e**) E-heating behaviors of CNT yarn embedded in a fabric and (**f**) Application of CNT yarn in a pillow as decoration and E-heating.

Analysis of Figure 5a revealed the steady-state temperature under an applied voltage of 3.0 V pointed out a rapid (only within 1s) dissipation of heat (175 °C) of pristine CNT yarn, whereas acidification could prompt up this dissipation up to 242 °C and esterification enabled it to reach further at 262 °C. The temperature difference from 0 to 3 V was 150 °C for pristine CNTY and 236 °C for esterified CNT yarn was noticed. Esterification generated inter-tube cross-links in between CNTs which finally assisted in the fast movement of electrons resulting in better electro-thermal properties. Notably, Figure 5b depicted changes for maximum time-dependent temperatures of pure, functionalized, and Esterified CNT yarn under various applied voltages (0.5 ~3V), where esterified CNT yarn exposed the highest temperature for each particular voltage, far greater than the pristine one. For example, at only 3 V, the maximum temperature for esterified CNT yarn was about 260 °C, 42% higher than pristine CNT yarn 175 °C and 21% higher than acidified one, i.e., 242 °C. To further explore this superb electro-thermal performance of esterified CNT yarn due to having ester bonds, we approached a cyclic test as shown in Figure 5c. A well-controlled variation in temperature, even after more than 45 on/off cycles, was noticed throughout the heating time of 15 min at a constant voltage of 3.0 V. Meanwhile, the compatibility of modified CNT yarn in a fabric through simple sewing as exemplified in Figure 5d, validated its brilliant flexibility to be used in E-textiles. In addition, the rapid increment of temperature from ambient to 20 °C, 30 °C, and 45 °C, respectively, to voltages of 1 V, 2 V, and 3 V, only in 5 s (Figure 5e) just interpreted the excellent efficacy of our bio-inspired CNT yarn for multifunctional applications, along with enabling it to be a promising candidate for open-air environment heaters (Figure 5f).

The esterified CNT yarn has shown high heating temperature at low trigger voltage with a quick response time, showing outstanding electro-thermal properties compared with other heating materials due to its inherited and crosslinked structure as shown in Table 1. The esterified yarn with covalent bridge between CNTs results in higher E-heating properties than other non-covalently bonded CNTs yarn, i.e., CNT with PDMS and CNT-Ppy-cotton [55,56].

### 3.5. Multifunctional Applications

**Figure 6:**
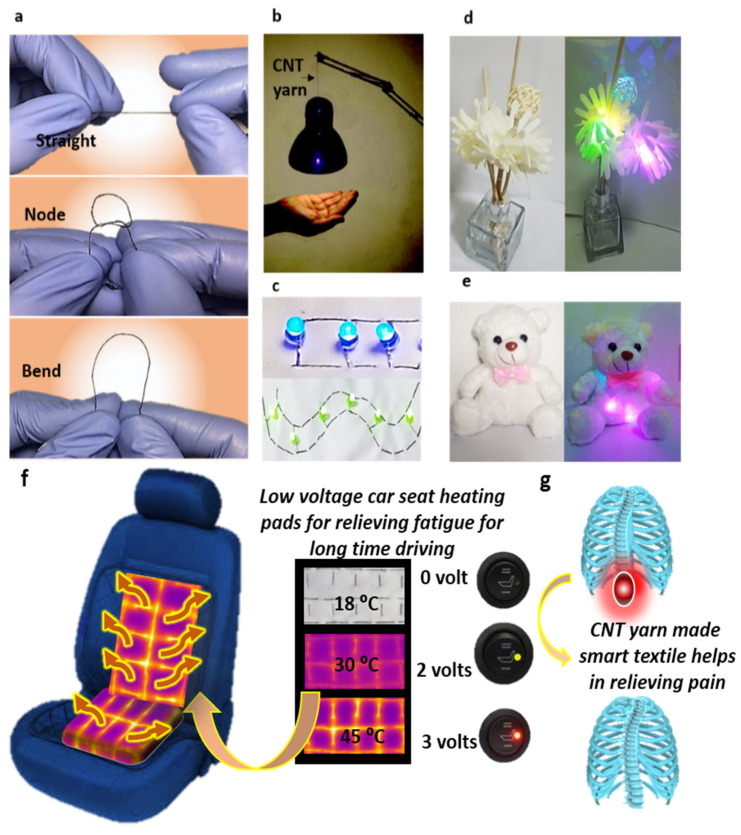
(**a**) Showing flexibility of cross-linked CNT yarn, i.e., straight, node, and bend; (**b**) Application of esterified CNT yarn as a conductor in hanging lamp; (**c**) Demonstrating sew-ability of CNT yarn into the fabric with LED; (**d**,**e**) Practical application of CNT yarn in home decoration; (**f**) Application of CNT yarn as a car seat heating pads with low supplied voltages; and (**g**) Showing relief of muscle.

Esterified CNT yarn has numerous daily life applications with excellent durability along with fundamental characteristics such as high electrical and electro-thermal conductivities in usage for home decoration, lamps, and seat heating pads, as shown in Figure 6. Cross-linked CNT yarn has the capability which can be easily sewn into woven, knitted, or non-woven fabric, which provided a great opportunity to a researcher for designing smart textiles according to need. Figure 6a showed the flexibility of CNT yarn in terms of straight, node, and bend position. Figure 6b amplified the E-heating performance showing the application of esterified CNT yarn as a conductor in a hanging lamp. Figure 6c–e demonstrated the sewability of CNT yarn into the fabric with LED so it could be used in decoration. Here, we prepared a prototype application of esterified CNT yarn in a car seat heating pad with low voltages as given in Figure 6f–g. Our modified CNT yarn possesses quick response E-heating features due to cross-linked CNTs, which could potentially relieve fatigue pain and muscles sore from particularly long driving time at low voltage supply.

## 4. Conclusions

In this study, an effective strategy was used to successfully develop a cross-linked CNT yarn through functionalization and esterification; fundamentally stimulated by the hierarchical structure of sarcoplasmic reticulum muscles. We could improve both electrical and electro-thermal properties of CNTs magnificently at a macroscopic level. The experimental observations showed much conductivity (~1950 S/cm) of esterified CNT yarn compared to untreated CNT yarn. Additionally, the electro-thermal performance of CNT yarn was also improved due to the formation of a covalent bond, resulting in a higher steady temperature of cross-linked CNT yarn, i.e., 261 °C at 3 V, much higher than pristine CNT yarn (175 °C). The modified CNT yarn also shows stable sensing behavior with mor than enough durability in strain sensing results. The cross-linked CNT yarn shows stable E-heating and sensing properties. To further elaborate these results, stable strain sensing properties of CNT yarn should be further investigated to monitor different deformations during various body postures. Taken together, the current paradigm of CNT yarn opens new possibilities and has the potential to be applied in versatile directions such as intelligent devices, smart clothing, or other advanced composites.

## Figures and Tables

**Table 1:** E-heating properties and performance of classic heating materials.

Materials	Process	Structure	Voltage (V)	Temp. (°C)	Time (s)	Ref.
CNT/film/PDMS	Coating	Composite	2	150	~30	[55]
CNT fiber	Twisting CNT ribbons	Yarn	5	135	<5	[57]
CNT-Ppy-cotton	Coating + Immersion	Yarn	5	45		[56]
CNT-I-PPy	Coating + Immersion	Yarn	3.5	75	16	[56]
Graphene fiber	Twisted ribbon	Yarn	6.6	44	<2	[58]
CNT	Twisting CNT socks	Rope	15 V	1000<		[59]
CNT/PDMS	Spray Coating	Composite	110	200	40	[60]
CVD CNT	Twisting	Yarn	3	175	<5	This work
Esterified CNT	Immersion + Twisting	Yarn	3	262	<5	

## Data Availability

Not applicable.

## Supplementary Materials

The following are available online at https://www.mdpi.com/article/10.3390/nano12020208/s1, Figure S1: TEM of pristine CNT (**a**) and functionalized CNT (**b**) and Figure S2: TGA of CNT yarn in Nitrogen Supply.
