# Supplementary material for: Bio-Inspired Hierarchical Carbon Nanotube Yarn with Ester Bond Cross-Linkages towards High Conductivity for Multifunctional Applications
Source: Nanomaterials (Basel). 2022 Jan 10;12(2):208. doi: 10.3390/nano12020208 (PMC8779581; doi:10.3390/nano12020208)
Supplement: Supplementary file 1 [file nanomaterials-12-00208-s001.zip › nanomaterials-1505078-supplementary.pdf]

Supporting Information

Bio-Inspired Hierarchical Carbon Nanotube Yarn with Ester Bond Cross-Linkages towards High Conductivity for Multifunctional Applications

Sidra Saleemi [1,2], Mohamed Amine Aouraghe [1], Xiaoxiao Wei [1], Wei Liu [3], Li Liu [1], M. Irfan Siyal [4], Jihyun Bae [5,*] and Fujun Xu [1,*]

[1] Key Laboratory of Textile Science & Technology, Ministry of Education, College of Textiles, Donghua University, Shanghai 201620, China; sidraatif.tet@pu.edu.pk (S.S.); med.aouraghe@gmail.com (M.A.A.); 1189138@dhu.edu.cn (X.W.); liliull@dhu.edu.cn (L.L.); fjxu@dhu.edu.cn (F.X.)
[2] Institute of Polymer and Textile Engineering, University of the Punjab, Quaid-e-Azam Campus. P.O. Box, Lahore 54590, Pakistan
[3] School of textiles and fashion, Shanghai University of Engineering Science, Shanghai 201620, China;
littlewand@163.com
[4] Department of Textiles and Clothing, National Textile University, Karachi Campus, *Karachi* 74900,
Pakistan; irfan.tex@gmail.com
[5] Human-Tech Convergence Program, Department of Clothing and Textiles, Hanyang University, Seoul 04763, Korea
* Correspondence: jbae2@hanyang.ac.kr (J.B.); fjxu@dhu.edu.cn (F.X.)

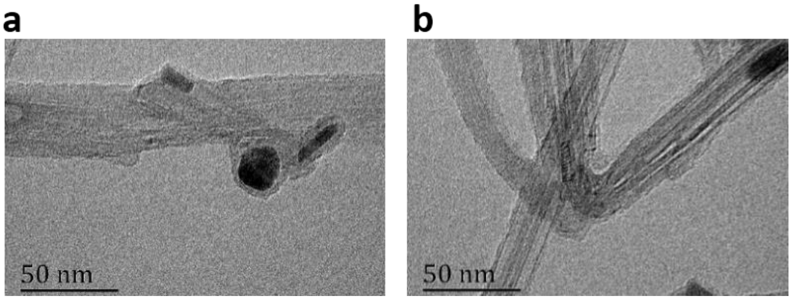

Figure S1. Please add a separate caption.

Figure S1 TEM of pristine CNT (a) and functionalized CNT (b) amorphous carbon was removed after the acid treatment. The functionalized CNT sample shows a wavy or bumpy and irregular surface, presenting functional groups which could be attributed to the esterification of CNTs [25,26].

The electrical conductivity (σ) of CNT yarns is calculated as:

$$\sigma = L / RA \text{ (S cm}^{-1}\text{)}$$

Where R, A, and L are the resistance, cross-sectional area, and length of the specimen.

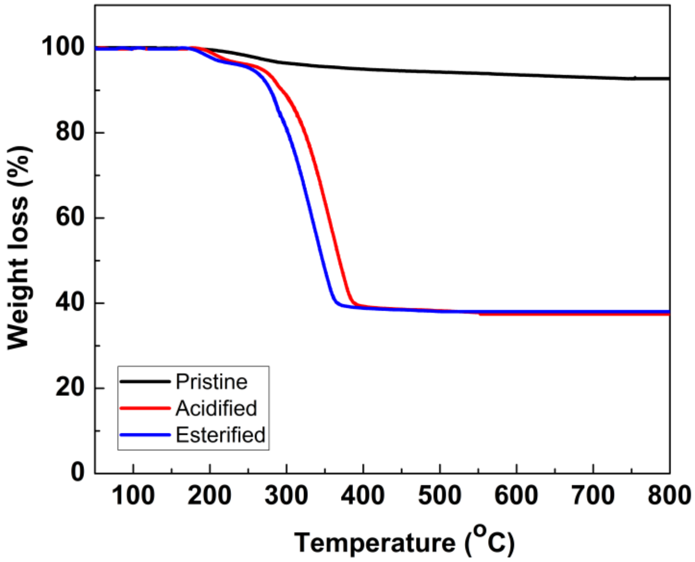

Figure S2. TGA of CNT yarn in Nitrogen Supply.

Figure S2 evaluated thermal gravity analysis (TGA) under nitrogen to assess the degradation behavior of CNT yarns for conductive applications. As chemical treatment caused defects (functional groups) on stable structure of CNTs thus results in early degradation [37]. TGA results verified that pristine CNT yarn remained stable in nitrogen supply up to 800 °C while treated CNT yarn started degradation at 280 °C.
